# Understanding the redox process upon electrochemical cycling of the P2-Na_0.78_Co_1/2_Mn_1/3_Ni_1/6_O_2_ electrode material for sodium-ion batteries

**DOI:** 10.1038/s42004-020-0257-6

**Published:** 2020-01-22

**Authors:** Charifa Hakim, Noha Sabi, Le Anh Ma, Mouad Dahbi, Daniel Brandell, Kristina Edström, Laurent C. Duda, Ismael Saadoune, Reza Younesi

**Affiliations:** 1 Materials Science and Nano-engineering, Mohammed VI Polytechnic University, Lot 660-Hay Moulay Rachid, Ben Guerir, Morocco; 2 LCME, Faculty of Science and Technology, Cadi Ayyad University, Av. A. El Khattabi, P.B.549, Marrakesh, Morocco; 3 Department of Chemistry – Ångström Laboratory, Uppsala University, Box 538, 751 21 Uppsala, Sweden; 4 Department of Physics and Astronomy, Division of Molecular and Condensed Matter Physics, Uppsala University, Box 516, 751 20 Uppsala, Sweden

**Keywords:** Batteries, Materials for energy and catalysis

## Abstract

Rechargeable sodium-ion batteries have recently attracted renewed interest as an alternative to Li-ion batteries for electric energy storage applications, because of the low cost and wide availability of sodium resources. Thus, the electrochemical energy storage community has been devoting increased attention to designing new cathode materials for sodium-ion batteries. Here we investigate P2- Na_0.78_Co_1/2_Mn_1/3_Ni_1/6_O_2_ as a cathode material for sodium ion batteries. The main focus is to understand the mechanism of the electrochemical performance of this material, especially differences observed in redox reactions at high potentials. Between 4.2 V and 4.5 V, the material delivers a reversible capacity which is studied in detail using advanced analytical techniques. In situ X-ray diffraction reveals the reversibility of the P2-type structure of the material. Combined soft X-ray absorption spectroscopy and resonant inelastic X-ray scattering demonstrates that Na deintercalation at high voltages is charge compensated by formation of localized electron holes on oxygen atoms.

## Abstract (web summary)

The inclusion of nickel and manganese in layered sodium metal oxide cathodes for sodium ion batteries is known to improve stability, but the redox behaviour at high voltage is poorly understood. Here in situ X-ray spectroscopy studies show that the redox behaviour of oxygen anions can account for an increase in specific capacity at high voltages.

## Introduction

Among the alternatives to lithium-based battery chemistries, the sodium-ion battery (SIB) technology has remained in the research focus due to its potential to decrease the cost^1,2^. Sodium and lithium both belong to the alkali metal group in the periodic table and thereby share common characteristics such as having the same valence state and they can be inserted or intercalated in layered oxides^3,4^. However, the larger ionic radius of sodium cation in comparison to lithium cations is accompanied with a lower energy density. Today, there is also a struggle to handle the instability of the crystal structures of typical cathode materials during sodiation/desodiation^5^.

Similarly, to the ongoing research on Li-ion batteries, there is a large variety of cathode materials being investigated for SIBs. In this context, sodium layered oxides (Na_x_MO_2_, (M = TM = transition metal)) have attracted significant attention thanks to their ease of synthesis, high capacity and good rate capability. Therefore, cathode materials such as Na_x_Co_y_Mn_1–y_O_2_^6,7^_,_ Na_x_Co_y_Ti_1–y_O_2_^8,9^, and Na[Fe_x_Mn_y_Ni_z_]O_2_^10,11^ have been extensively studied in literatures.

The Na_x_MO_2_ layered oxides are built up of sheets of edge sharing metal oxide octahedra (MO_6_), with Na ions located between these layers. According to Delmas’s nomenclature, sodium based layered materials can be categorized into two main groups: P2-type (0.6 < *x* < 0.7, where *x* = sodium stoichiometry) and O3-type (*x* ≈ 1). In these structures the sodium ions occupy the prismatic sites (P) or the octahedral sites (O), respectively^12^, and the number (*n* = 2 or 3) refers to the repeat period of the transition metals stacking. P2 and O3 type structures differ in their oxygen stacking sequences: ABBA for the P2 type and ABCABC for the O3 type.

The diffusion mechanism of Na^+^ in the P2 type is different than that in the O3 type. Although the O3 type structure has a high sodium content, the diffusion of the sodium from one octahedral site to another occurs through face-shared interstitial tetrahedral sites, which generate a comparatively slow diffusion^13^. The P2 type structure on the other hand has an open path for sodium ions, where they diffuse through an open square surrounded by four oxide ions^3^. However, the sodium deficiency in the P2 type structure leads to significant reduction of the delivered capacity when a sodium-free anode material, such as hard carbon, is used in a full-cell setup. Use of sacrificial salt to compensate the sodium deficiency has therefore been demonstrated to compensate for this shortcoming^14,15^.

Moreover, P2-type cathodes suffer from capacity fading when operated in a high voltage range, which in turn result in a shortened cycle life. It has been suggested that oxygen release as well as the cation migration associated with the oxygen activity at high voltages could be the reason behind this capacity loss^16^. At the same time, other researchers have shown that cathode materials with higher capacity can be realized by optimizing and controlling the oxygen activity, since charge can be stored in both transition metals and oxide ions^17^.

A large number of papers have discussed the oxygen activity in Li-excess cathode materials in lithium-ion batteries they believed that the introduction of alkali ions into the transition metal layers result in relatively ionic Li^+^-O2p-Li^+^ interactions, which places these O2p at the top of O-valence band and then promote oxygen redox activity in these materials^18–21^. Correspondingly, anionic redox activity in sodium ion batteries was rarely reported. However, it can also be expected. Using electron energy loss spectroscopy (EELS) and soft X-ray absorption spectroscopy (sXAS), Ma et al. demonstrated the existence of oxygen vacancies at the surface of Na_0.78_Ni_0.23_Mn_0.69_O_2_ at high voltages, which suggests the participation of oxygen anions in the charge compensation mechanism, thereby resulting in considerably higher capacity than the theoretical value^22^. Maitra et al. reported that Na_2/3_[Mg_0.28_Mn_0.74_]O_2_ exhibits oxygen redox activity, which displays that excess of alkali metal is not a requirement for oxygen redox activity. Furthermore, the presence of Mg^2+^ in the TM layers suppress the undesired oxygen loss^23^. This effect was further supported by the existence of both oxygen redox activity as well as absence of O_2_ release in alkali-deficient Na_2/3_Mn_7/9_Zn_2/9_O_2_, despite evidence of cation migration observed upon cycling. This was observed by Bai et al. who could see a tendency of Zn to migrate into the more favorable tetrahedral site, while these sites remains less favorable for Mn^24^.

The aim of this study is to understand the mechanism of redox activity of the P2-type cathode material Na_0.78_Co_1/2_Mn_1/3_Ni_1/6_O_2_. This composition was selected following extensive investigations performed by Doubaji et al.^25^, which showed a positive impact of cobalt substitution by Ni and Mn in terms of structural stability and electrochemical performance. The redox process at high voltages was, however, not clarified in these studies.

Herein, we investigate the redox mechanisms upon the charge/discharge process of a Na//Na_0.78_Co_1/2_Mn_1/3_Ni_1/6_O_2_ electrochemical cell by using several advanced analytical tools such as in situ X-Ray diffraction (XRD), X-ray absorption spectroscopy (XAS), and resonant inelastic X-ray scattering (RIXS), which elucidate the participation of the anions redox reactions to compensate the sodium deintercalation at high voltages.

## Results and discussion

### Characterization of the structure and morphology

The elemental composition of P2-Na_x_Co_1/2_Mn_1/3_Ni_1/6_O_2_ measured by ICP-AAS measurements showed the molar Na:Co:Mn:Ni ratio of 0.78:0.503:0.331:0.165. The ratio between the transition metals is thus consistent with expected stoichiometry, however, the high content of sodium observed is likely originating from the excess of sodium used during synthesis.

**Fig. 1:**
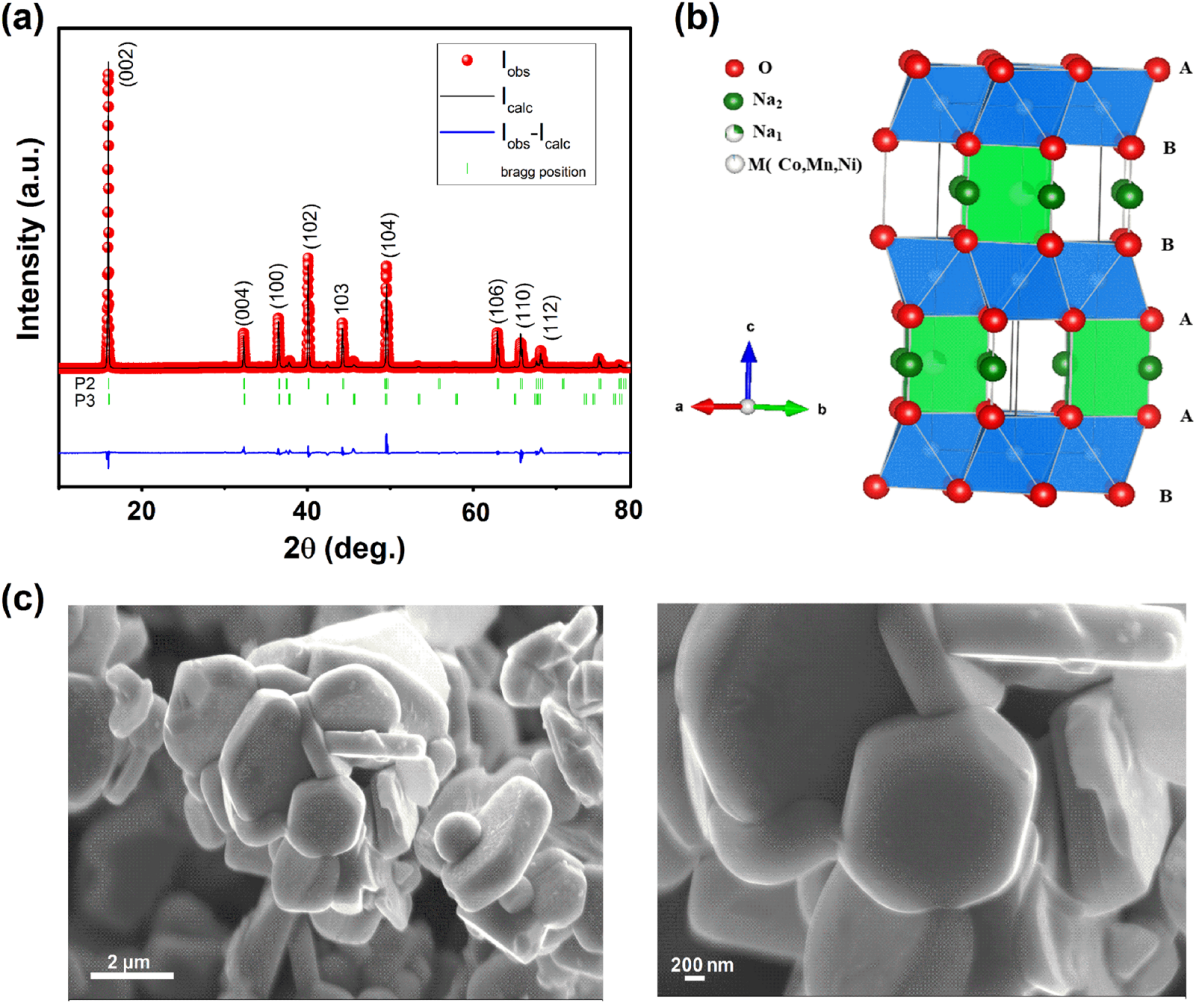
Structural and morphological study of P2-Na_0_._78_Co_1/2_Mn_1/3_Ni_1/6_O_2_ powder. **a** Rietveld refined XRD patterns. **b** Schematic illustration of P2-structure. **c** SEM micrographs taken at different magnifications.

Figure 1a shows the XRD pattern of the pristine sample. All the intense diffraction peaks are indexed to a hexagonal lattice with space group *P*_63/mmc_, and additional small peaks can be indexed to a P3-type structure (space group: R3m). It is clear that the diffraction lines of the P2-type are much stronger than those of the P3-type, which is also illustrated from the Rietveld ratio (P2: 96 wt%, P3: 4 wt%). The cell parameters obtained (*a* = 2.83857 Å, *c* = 11.06986 Å) are in close proximity to those reported for P2-Na_x_Co_2/3_Mn_2/9_Ni_1/9_O_2_: *a* = 2.8274 Å, *c* = 11.0553 Å^25^. The different parameters obtained by Rietveld refinement for P2-type structure are presented in supplementary Table 1. A schematic illustration of the P2-type structure using the Vesta program for Na_0.78_Co_1/2_Mn_1/3_Ni_1/6_O_2_ is presented in the Fig. 1b, Cobalt, manganese and nickel ions are located in octahedral sites while Na ions are coordinated by oxygen in two trigonal prismatic sites: Na_e_, where Na is sharing edges with six surrounding (Co, Mn, Ni)O_6_ octahedra, and Na_f_, where Na shares faces with two neighbor (Co, Mn, Ni)O_6_ octahedra.

The SEM micrographs of Na_0.78_Co_1/2_Mn_1/3_Ni_1/6_O_2_ are shown in Fig. 1b. The sample exhibits hexagonal-shaped primary particles with an average size of 1–2 µm, which reflects the material’s hexagonal symmetry. It can also be seen that these primary particles undergo agglomeration to form secondary particles which are several micrometers.

### Electrochemistry

**Fig. 2:**
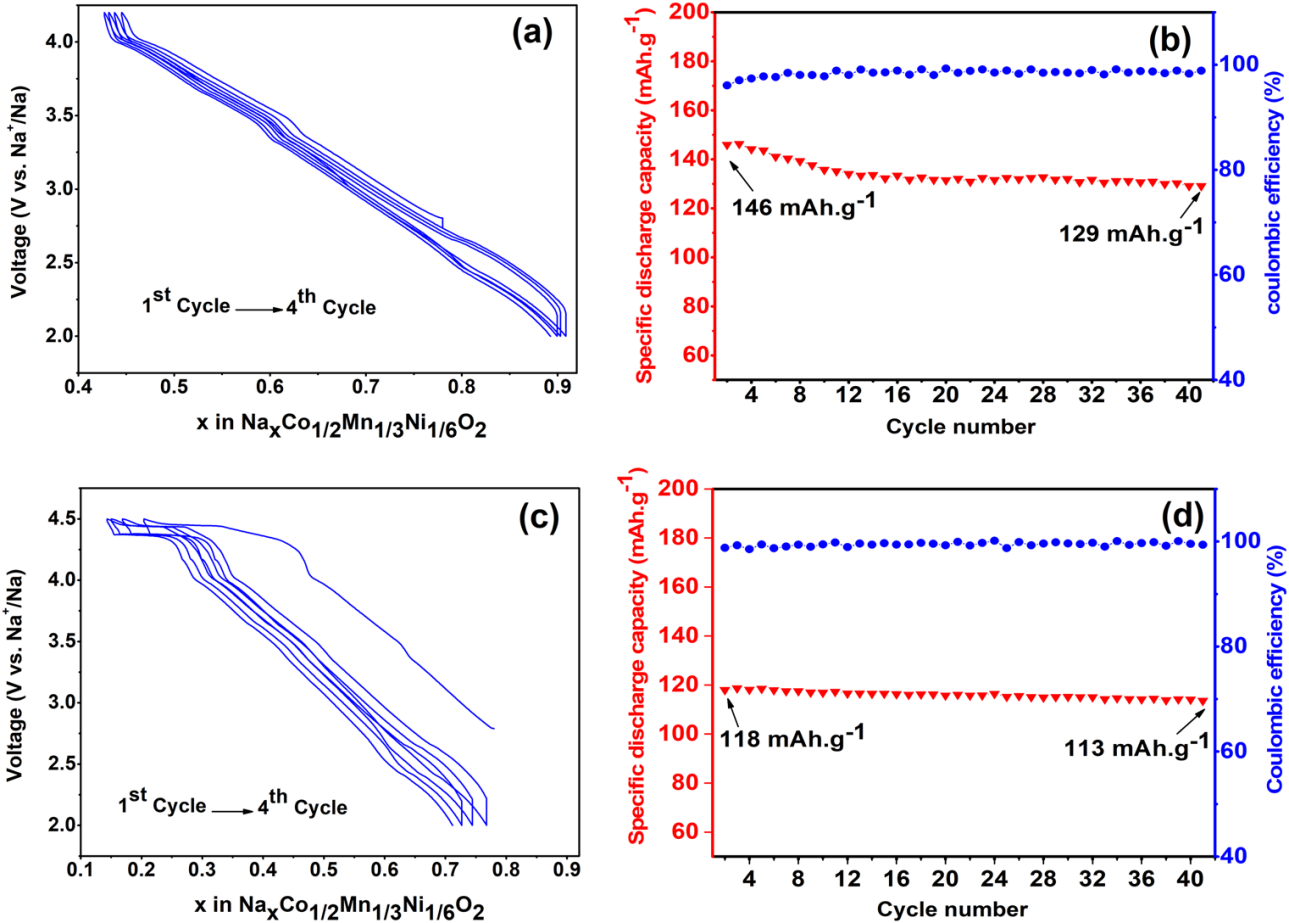
Electrochemical performances of P2-Na_0.78_Co_1/2_Mn_1/3_Ni_1/6_O_2_ at two different potential windows. **a**, **b** Cycling performances in a potential window of 2–4.2 V. **c**, **d** Cycling performances in a potential window of 2–4.5 V.

The electrochemical performance of Na_0.78_Co_1/2_Mn_1/3_Ni_1/6_O_2_ was tested in room temperature using sodium half-cells. The open circuit voltage (OCV) value was about 2.7 V (vs. Na^+^/Na) for all tested cells. Figure 2 displays galvanostatic cycling results of P2- Na_0.78_Co_1/2_Mn_1/3_Ni_1/6_O_2_ electrodes using two different cut-off potentials of 4.2 V and 4.5 V, respectively using a current density of 25 mA/g.

Figure 2a shows that the first charge capacity is equal to 85 mAh g^−1^ corresponding to 0.334 Na^+^ when the cell is charged to the cut-off potential of 4.2 V. In the following discharge process, the capacity is about 118 mAh g^−1^, meaning that the Coulombic efficiency of the first cycle is above 100%. This means that the amount of Na in the cathode formulation has increased from 0.78 to 0.89 Na^+^ after the first cycle. This is due to the fact that P2-type materials contain sodium vacancies and when cycled vs. metallic sodium, a larger amount of sodium can be inserted into the structure. The electrochemical profiles of the subsequent 40 cycles almost completely overlap and the cathode material delivers capacities between 118 to 113 mAh g^−1^ with a retention of above 95% and a very promising Coulombic efficiency exceeding 99.3% (see Fig. 2b).

Figure 2c shows the galvanostatic charge/discharge voltage profiles when the high cut-off potential is increased to 4.5 V. The cathode material delivers a higher capacity compared to the cell with cut-off potential of 4.2 V; for the first charge and discharge the capacities of about 146 mAh g^−1^ and 143 mAh g^−1^, respectively, were achieved. These capacities correspond to removal of 0.58 Na^+^ and addition of 0.56 Na^+^ during the first charge and discharge, respectively. The extra capacity obtained is due to the appearance of a new and relatively flat high voltage plateau (above 4.2 V) which correspond to an additional capacity of 25 mAh g^−1^. The discharge capacities decreased after the first cycle, with a capacity retention of 88.3% and a coulombic efficiency of 98.8% after 40 cycles (Fig. 2d).

The flat plateau between 4.3 and 4.5 V indicates that a reaction occurred at high potential, which is fundamentally different from the charge plateau observed in narrow potential windows up to 4.2 V. This plateau is longer in the first charge, but it provides some reversible capacity from the second cycle. There are several mechanism that can explain the origin of this high voltage plateau: (i) phase transition such as from P2 to O2^26,27^, or from P2 to OP4^28,29^, or P2 to Z phase^30^ (ii) redox activity of oxygen anions^22,31^, or (iii) electrolyte decomposition on at the surface of electrode at high potentials^32^.

The latter is not the possible explanation for the extra capacity at high potentials since cyclic voltammetry and XPS spectra showed no indication of electrolyte decomposition. The C 1s, F 1s, and O 1s spectra of electrodes analyzed at different state of charge during the first cycle look very similar to the pristine electrode, implying absence of electrolyte degradation products (see supplementary Fig. 1). To understand whether the first two mechanism explain the origin of the plateau at high potentials, results from in situ XRD, XAS, and RIXS measurements of Na_0.78_Co_1/2_Mn_1/3_Ni_1/6_O_2_ electrodes are discussed below.

### Structural evolution of P2-Na_0.78_Co_1/2_Mn_1/3_Ni_1/6_O_2_ during cycling

**Fig. 3:**
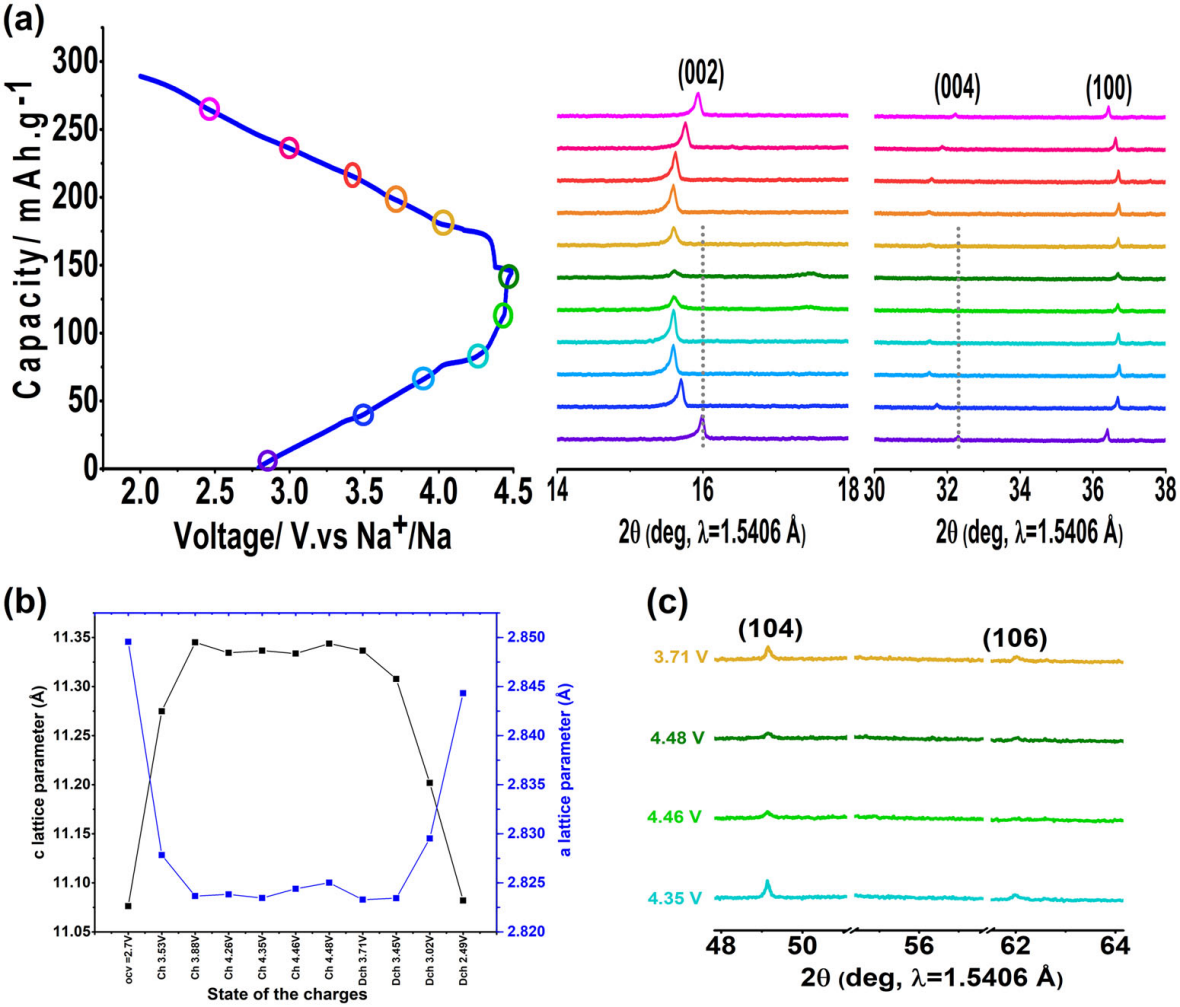
Evolution of P2-Na_0_._78_Co_1/2_Mn_1/3_Ni_1/6_O_2_ structure during cycling using in situ XRD. **a** Voltage–time curve of the first cycle at 0.1C rate in the potential range of 2–4.5 V and the magnifications of 002, 004, 100 in situ XRD reflections during cycling**. b** The evolution of the hexagonal unit cell parameters during the first cycle obtained from Rietveld refinement. **c** extended view of the 104 and 106 reflections at high potentials.

A phase transition is known to be associated with a volume change in the material structure, which contributes to structural irreversibility and leads to poor rate and cycling performances. The structural changes of P2-Na_0.78_Co_1/2_Mn_1/3_Ni_1/6_O_2_ during the first cycle in the voltage range of 2–4.5 V was monitored using in situ XRD. The voltage-time profile and selected in situ XRD patterns are plotted in Fig. 3. The overview of the diffraction patterns reveals that the major diffraction lines of the P2-type structure are maintained, which means that the material preserves its P2 structure during the entire electrochemical process.

Figure 3 shows that in the beginning of the charge process (00ℓ) and (10ℓ) reflections shift to lower angles, in contrast to (100) shifting to a higher angle (Fig. 3a). This indicates that the “*c*” lattice parameter increases, most likely due to the increase in the electrostatic repulsions between the oxygen layers upon sodium extraction. At the same time, the “*a*” lattice parameter which represents the M–M distance decreases. This is expected from the increase in the oxidation state of the transition metals (Co^3+^ → Co^4+^, Ni^3+^ → Ni^4+^), which is needed to maintain the neutrality of the material during sodium removal (Fig. 3b).

The XRD peaks become broader with lower intensity at high voltage, especially for the sample fully charged to 4.5 V as compared to at the beginning of the charge process. Simultaneously, the appearance and growth of a very weak peak between 17° and 18° can be seen, which is not observed in the in situ XRD pattern in the voltage range 2.0–4.2 V (see supplementary Fig. 2). These changes may be associated with the formation of O2 stacking faults (i.e., changes in the evolution of *a* and *c* lattice parameters). Indeed, the sodium ions in the P2-type structure occupy the prismatic sites between neighboring oxygen layers, and upon sodium extraction the MO_2_ slabs shift into the a–b plane to avoid oxygen–oxygen contacts. There are two possible directions for these shifts to form the O2-type structure. The stacking faults are formed because these two choices are selected randomly, which means that any long range ordered structure is lost^33^. The broadening of the (10ℓ) peaks (e.g., (104) and (106)) confirms the presence of these stacking faults in Na_0.78_Co_1/2_Mn_1/3_Ni_1/6_O_2_ (Fig. 3c)^34^.

The reverse process occurred during discharge: the well-defined and sharp P2 peaks were slowly recuperated upon sodium insertion, which means that the stacking faults formed previously during charge are eliminated during discharge. This is primarily owing to the incapability of the P2-type structure to accommodate stacking faults between adjacent MO_2_ layers, unlike the O2-type. Regardless of these variations during cycling, the material shows a good structural reversibility of the P2 type structure in the investigated voltage range.

### XAS and resonant inelastic X-ray scattering measurements

**Fig. 4:**
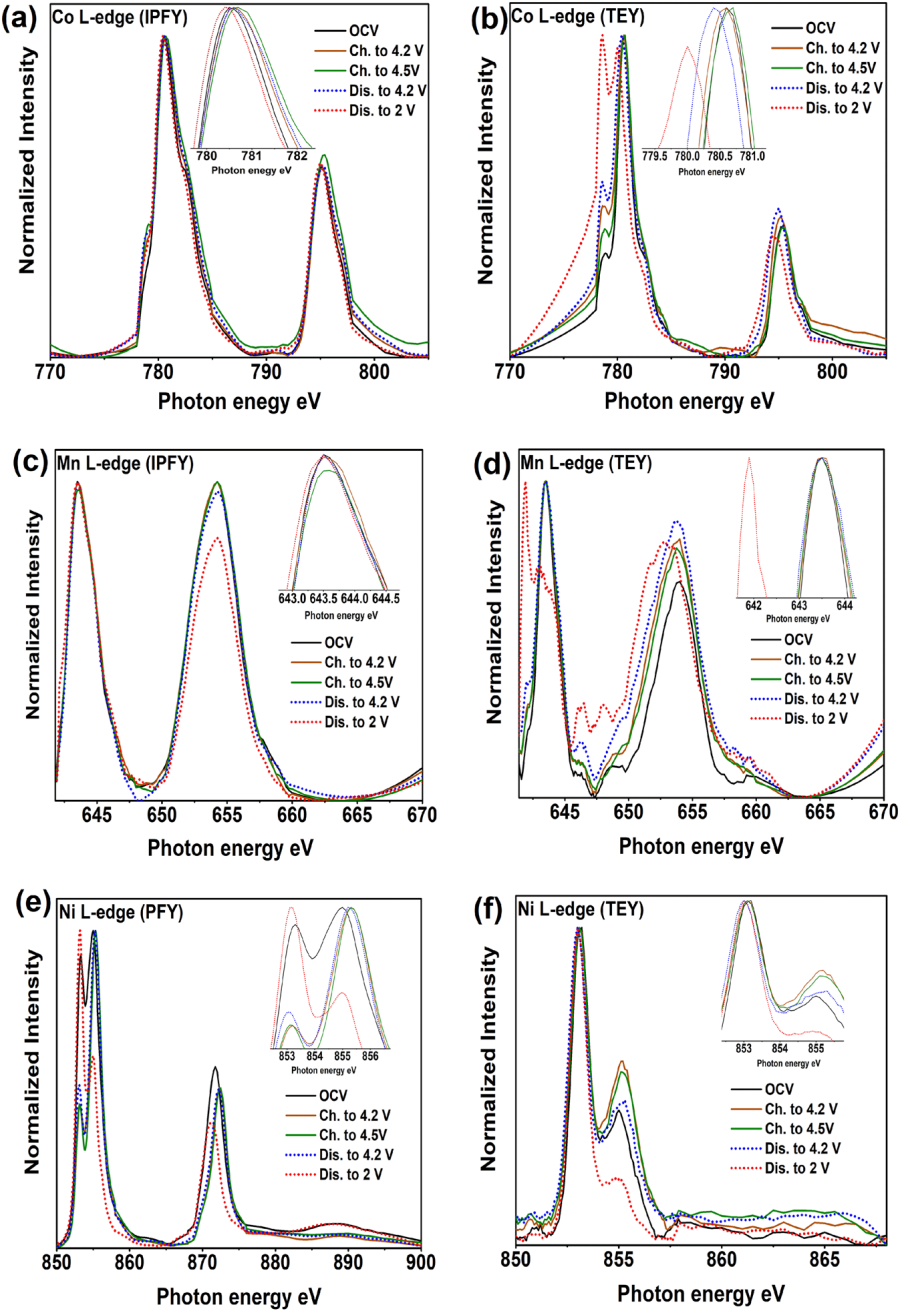
L_II,III_ X-ray absorption spectroscopy spectra collected at different states of charge. **a** Co in PFY mode. **b** Co in TEY. **c** Mn in IPFY. **d** Mn in TEY. **e** Ni in PFY. **f** Ni in TEY mode. Insets: extended view of L_3_ peaks. (states of charges: OCV, charged to 4.2 V, charged to 4.5 V, discharged to 4.2 V, discharged to 4.5 V).

The electronic structure of Co, Ni, Mn (L_2,3_-edge), and O (K-edge) during the first sodiation/desodiation was analyzed by a range of spectroscopic techniques, including soft XAS using different modes with different probing depths; total-electron yield (TEY) (surface sensitive), partial-fluorescence-yield (PFY), and inverse partial-fluorescence-yield (IPFY) (bulk sensitive)^35^, and RIXS measurements. The results provide information regarding the redox reactions taking place upon cycling as well as the hybridization states for metal-oxygen octahedral units. Figure 4 shows XAS spectra of nickel, cobalt and manganese L_2,3_-edges collected ex situ on electrodes at different state of charge.

The Co L_2,3_-edge XAS corresponds to the transitions from the Co 2*p*_3/2_ and 2*p*_1/2_ core electrons, split by the spin–orbit interaction of the Co 2*p* core level, to the empty 3*d* orbitals. The profile and the peaks positions of the pristine spectrum obtained in both PFY and TEY modes (Fig. 4a, b) are very similar to those reported for LiCoO_2_ and thus clearly demonstrates that cobalt in the pristine material is in its trivalent state^36^. The spectra in PFY mode show that during charge, the main peak broadens and shifts slightly towards higher energy by 0.2 eV from the pristine to the fully charged material at 4.5 V, which suggests oxidation of Co^3+^ to Co^4+^. It has previously been claimed that the small peak shift is due to the low participation of cobalt in charge compensation process during charge^37–40^. The shift is reversible during discharge. However, in TEY mode the peaks of fully discharged electrode clearly shift toward lower energy, which is a much bigger shift compared to that in PFY mode, suggesting that the reduction of Co^3+^ to Co^2+^ mainly occurs at the surface of the material.

The Mn L_2,3_-edge spectra of the pristine electrode show a main peak at 643.4 eV and a sub-peak at 640.7 eV, which coincident with that of the Mn^4+^. No peak shift was observed during the charge process in either TEY or IPFY modes, indicating that Mn stays at tetravalent state and does not participate in the charge compensation during sodium deintercalation. The oxidation state of Mn seems unchanged during the discharge process in the IPFY mode, while the shift of the main peak for the fully discharged electrode is clearly seen in the TEY mode and reveal the reduction of Mn^4+^ at the surface.

Figures 4e, f show the Ni-L edge spectra taken in both PFY and TEY modes. Ni L_2,3_-edge XAS of the pristine electrode obtained in PFY mode shows that the Ni exist as Ni^3+^ in the bulk^41^. Nickel is oxidized to Ni^4+^ during sodium removal when charging the material up to 4.2 V, and no shift of the main peak was observed from 4.2 to 4.5 V. By discharging to 2 V, the spectrum almost returns to the same position of the pristine material but with different intensity ratio between L_3high_ and L_3low_ suggests the reduction to Ni^2+^. In contrast, the spectra in TEY mode shows that the Ni is mainly in its divalent state in the surface layer while it is oxidized during charging to 3+ and returns to its previous oxidation state during discharge. The transition metal oxidation gradient between the surface and the bulk of the material has been seen in previous studies and was then attributed to structural changes at the surface^42–44^.

**Fig. 5:**
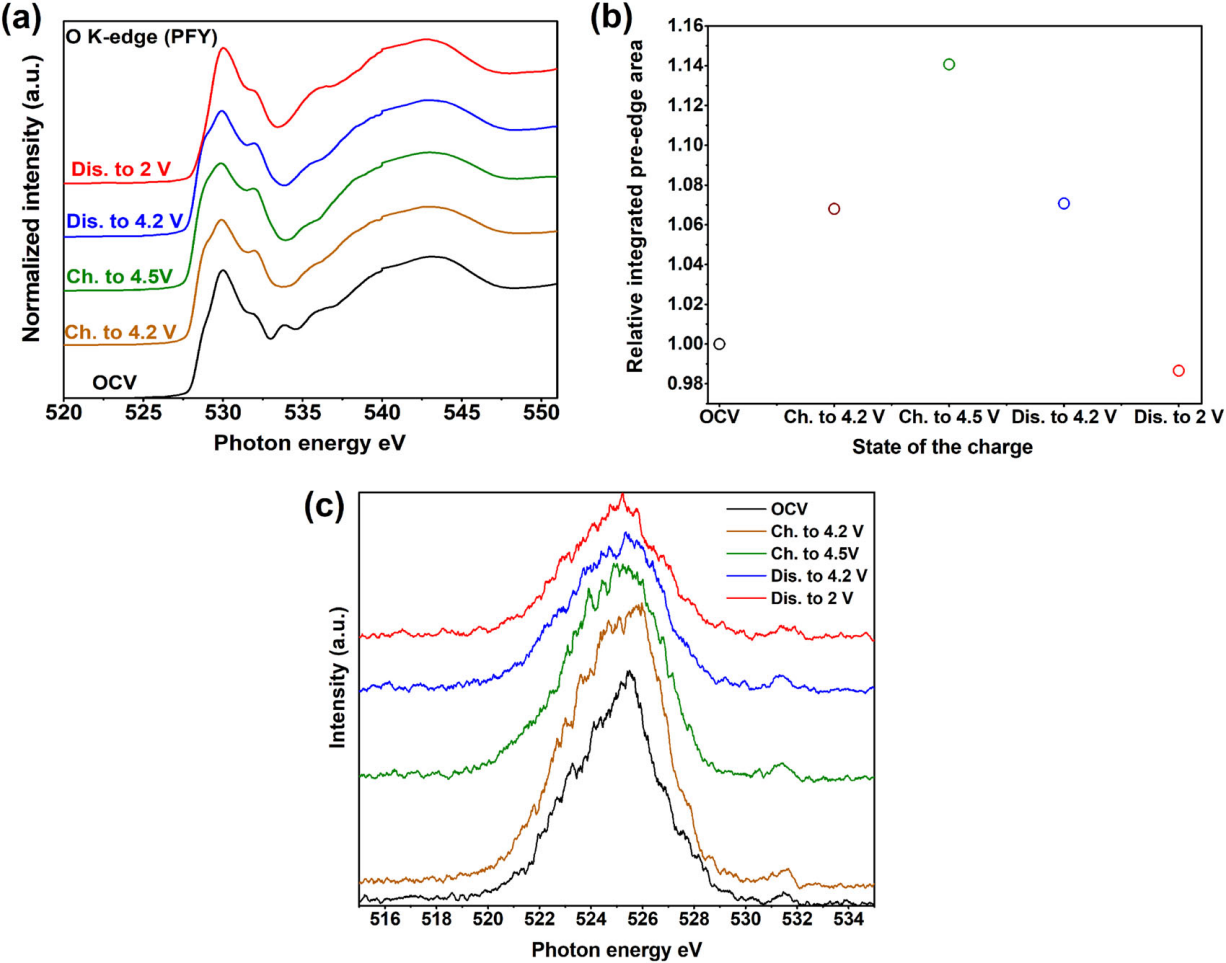
Investigation of the oxygen electronic structure. **a** Oxygen K-edge XAS spectra collected at different states of charge in partial fluorescence yield mode. **b** Variation of the integrated intensity in the low-energy region (≤534 eV) for O K-edge SXAS. **c** RIXS on the O K-edge of Na_0.78_Co_1/2_Mn_1/3_Ni_1/6_O_2_ with an excitation energy of 531.5 eV.

The O K-edge XAS spectra of Na_0.78_Co_1/2_Mn_1/3_Ni_1/6_O_2_ electrodes at different state of charge are shown in Fig. 5a. The spectra can be divided into two regions. First, a broad feature at higher energy (≥535 eV) appears, which is associated with the electronic transitions from O 1*s* to O 2*p*-states that are hybridized with transition metal-4*sp* states. Second, the pre-edge peaks below 535 eV correspond to the transition from O 1*s* to O 2*p*-states that are hybridized with transition metal-3*d* states, which is discussed in the following. The peak around 534 eV might be associated with some carbonaceous components that exist on the surface of the material. The main contribution to the pre-edge peaks comes from transitions to Co^3+^ and Mn^4+^ states, as these are the dominant transition metal ions in the compound and accounts for 84% of the spectral transitions (see Supplementary Fig. 3 and Supplementary Note 1). Therefore, the pristine spectrum can be explained by a superposition of the O K-XAS peaks of LiCoO_2_ and MnO_2_^40,45^. Figure 5b illustrates the changes in the area under the spectra between 520 and 534 eV of Fig. 5a and is a measure of the changes in the density of hole states just above the Fermi level.

When charging the material up to 4.2 V, the increase in the density of the empty states by 6.8% is caused by the oxidation of Ni^3+^ and Co^3+^ in Na_0.78_Co_1/2_Mn_1/3_Ni_1/6_O_2_, which creates more holes in the 2*p*–3*d* hybrid orbitals_._ A continuous increase in the density of the empty states is observed across the plateau (between 4.2 and 4.5 V) by an additional 7.27%. This increase cannot be explained by the oxidation of Ni, because the Ni L_2,3_-edge XAS spectra show no shift of the main peak in this region. However, a small shift was observed in the Co L_2,3_-edge XAS spectra above 4.2 V which corresponds to 4.28% (see Supplementary Fig. 4). Therefore, additional unoccupied states are associated with the removal of electrons from oxygen 2*p* orbitals, showing the small participation of anions in the redox activities in the charge compensation process in the material. To examine the nature of the hole states on oxygen, O K-edge RIXS spectra were collected as a function of state of charge (Fig. 5c).

RIXS allows us to detect the emission from the O 2*p* valence states below the Fermi level. There is a clear change in the RIXS spectra at 4.2 and 4.5 V (high voltage plateau) compared to the pristine spectrum. A considerable broadening at the main peak occurred and the elastic peak at the 531.5 eV is growing in intensity, which indicates the creation of hole states localized on the oxygen at high voltage^17,20^. However, if we compare the RIXs-spectrum of the OCV with the fully discharged electrode, a change in spectral weight is observed, suggesting lattice oxygen rearrangement during the first cycle.

Based on these results, we showed that electrons from oxygen atoms can participate in the charge compensation process during sodiation–desodiation and give rise to the excess of the capacity observed across the plateau at high voltage in Na_0.78_Co_1/2_Mn_1/3_Ni_1/6_O_2_ with a high proportion of cobalt cations.

**Fig. 6:**
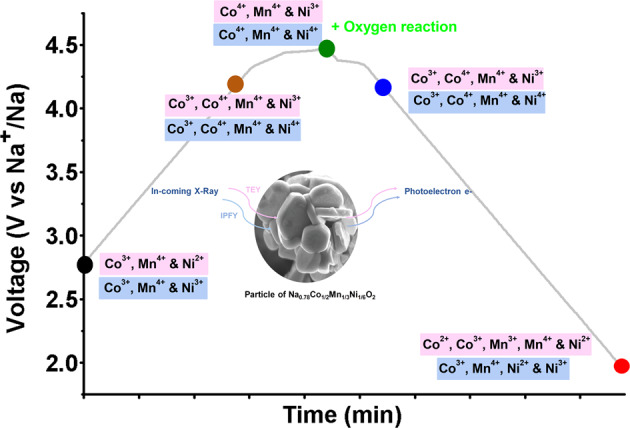
Schematic illustration to summarize the results of XAS and RIXS. Measurements showing the oxidation states of Ni, Mn, and Co in the bulk and surface.

In summary, the Na_0.78_Co_1/2_Mn_1/3_Ni_1/6_O_2_ cathode material can provide an extra reversible capacity of about 25 mAh/g between 4.2 and 4.5 V, in addition to reversible and stable capacity of about 118 mAh/g obtained in the voltage range between 2.0 and 4.2 V. The mechanism of redox reactions and the origin of the extra capacity is analyzed using variety of in-house and synchrotron-based analytical techniques. In situ XRD and XPS results excluded major crystal structure deformation or electrolyte degradation as the origin of extra capacity at high potential between 4.3 and 4.5 V. On the other hand, XAS and RIXs measurements revealed that upon charging up to 4.5 V the Mn valency remains at +4 state, while Co and Ni ions are oxidized to Co^4+^ and Ni^4+^. The continuous increase in the density of the empty states from 4.2 to 4.5 V indicated the removal of electrons from oxygen 2*p* orbitals implying contribution of anionic redox activities in charge process. The main findings of this study are schematically presented in Fig. 6.

## Methods

### Preparation of the samples

P2-Na_0.78_Co_1/2_Mn_1/3_Ni_1/6_O_2_ was prepared by a co-precipitation method. A solution made of Co(NO_3_)_2_·6H_2_O, Ni(NO_3_)_2_·6H_2_O and Mn(NO_3_)_2_·4H_2_O (1 M of the transition metals), was dripped into a NaOH solution (1 M) under rapid stirring. After 3 h stirring at room temperature, the precipitate formed was filtered, washed three times with distilled water and dried at 180 °C over night in air to remove residual water. The dried powder was mixed and grinded with Na_2_CO_3_ in an agate mortar for 45 min at room temperature. A 10 wt% excess of Na_2_CO_3_ was added to compensate for sodium volatility at high temperature. The obtained mixture was heated to 850 °C at a rate of 2 °C min^−1^ for 12 h in air, followed by slow cooling to room temperature and thereafter stored in an Ar-filled glove box.

### Material characterization

The chemical composition of the Na_x_Co_1/2_Mn_1/3_Ni_1/6_O_2_ material was measured using inductively coupled plasma optical emission spectrometry (ICP-OES) (Agilent Technologies 5110). The structure of the synthesized material was characterized by XRD using a Bruker twin–twin [Cu Kα radiation, *λ* = 1.54056 Å] by measuring the diffraction angle (2*θ*) between 10° and 80° in a continuous scanning mode with a step size of 0.01°. XRD data were analyzed by the Rietveld method using FULL-PROF software. The morphology of the pristine material and the size distribution of the particles were measured by scanning electron microscopy using a Zeiss Leo 1550 scanning electron microscope.

### Electrochemistry

Electrodes were fabricated by mixing 80 wt% of active material with 15 wt% carbon black and 5 wt% of polyvinylidene difluoride (PVDF) dissolved in N-methyl-2-pyrrolidone (NMP). The black slurry formed was coated on aluminum foil and was then dried at 80 °C for 4 h. Afterwards, electrodes were cut into 13 mm disks using a perforator and dried at 120 °C in a vacuum furnace inside the glovebox to remove any moisture or solvent contaminations. For cell assembly, the mass loading of the active material was approximately 2.1 mg cm^−2^, metallic sodium (Sigma-Aldrich) was used as both counter and reference electrodes. Whatman glass fiber membrane was used as the separator. The electrolyte was composed of 0.5 M of NaPF_6_ dissolved in a mixture of ethylene carbonate (EC) and diethyl carbonate (DEC) with a volume ratio of (1:1); 5 vol% of fluoroethylene carbonate (FEC) was added to improve the electrochemical performances. Cells were fabricated in CR2032 coin-type format and were galvanostatically cycled at room temperature using a current density of 25 mA/g. Two cutoff voltages of 2.0 and 4.2 V or 2.0 and 4.5 V, respectively, were used. All the potential values are hereafter reported vs. Na/Na^+^.

### In situ X-ray diffraction

In situ analysis were performed on Stoe and Cie Gmbh Stadi X-ray powder diffractometer equipped with Ge monochromator (CuK_α1_) using “coffee bag” (polymer laminated aluminum pouch) cells. XRD patterns were continuously collected in reflection mode with a movable detector around the fixed sample, within the 2*θ* range of 10–80°. The data collection time for each XRD scan was 20 min.

### X-ray photoelectron spectroscopy

Measurements were performed using a Scienta ESCA 300 instrument with the exciting radiation of Al Kα (hν = 1486.7 eV). The cells were disassembled in an argon-filled glove box, and the electrodes were thereafter washed with dimethyl carbonate (DMC) in order to remove excess electrolyte before the analysis. The samples were transferred into the introduction chamber of the XPS instrument using a special transfer cup to avoid any exposure to ambient air. The pressure in the analysis chamber was 1 × 10^−8^ mbar. The peak at 284.4 eV in the C 1*s* spectra was used as the reference to calibrate the binding energy scale. The spectra were analyzed using CasaXPS software.

### XAS and resonant inelastic X-ray scattering

Soft XAS as well as resonant inelastic X-ray scattering (RIXS) measurements were performed, using beamline BL27SU of SPring-8, Japan^46^ XAS spectra were collected for the Mn L-edge, Co L-edge, Ni L-edge, and the O K-edge by collecting energy-resolved fluorescence x-rays (500–1000 eV) and by measuring the sample drain current simultaneously. Bulk representative XAS spectra for Co L-edges, Ni L-edges, and the O K-edges are obtained by using the partial fluorescence yield (PFY) mode while the Mn L-edge could only be adequately obtained by using the inverse partial fluorescence yield (IPFY) mode (derived by inverting the O K-edge PFY). The surface-related drain current produces XAS spectra equivalent to the total electron yield (TEY) mode. The (I)PFY mode XAS spectra were collected by using an energy resolving, liquid nitrogen cooled, solid state detector. The TEY mode XAS spectra and the incident intensity were measured by recording the sample drain current and focusing mirror current using pico-ammeters. (I)PFY mode XAS spectra are bulk sensitive with an average information depth of about 100 nm while TEY mode XAS spectra are more surface sensitive with an average information depth of about 10 nm. The monochromator band width was set to ~0.2 eV (O K-edge) and ~0.3 eV (transition metal L-edges), respectively. Ni L3-edge and O K-RIXS measurements were performed using a high-resolution, variable line space grating spectrometer. The combined instrumental (monochromator and spectrometer) resolution ranged between about 0.3 eV for O K-RIXS and 0.5 eV for Ni L-RIXS.

## Supplementary information

Supplementary Information

## Data Availability

All data used in this manuscript are available from the authors upon reasonable request.
