# Supplementary material for: Understanding the redox process upon electrochemical cycling of the P2-Na0.78Co1/2Mn1/3Ni1/6O2 electrode material for sodium-ion batteries
Source: Commun Chem. 2020 Jan 22;3:9. doi: 10.1038/s42004-020-0257-6 (PMC9814369; doi:10.1038/s42004-020-0257-6)
Supplement: Supplementary file 1 — Supplementary Information [file 42004_2020_257_MOESM1_ESM.pdf]

| **P2 Structure** | |
|---|---|
| Space Group | $P6_3/mmc$ |
| Lattice Parameters | a= 2.83857 Å, C= 11.06986 Å, V=77.245 Å$^3$ |
| Percentage | 96.06% |
| $R_{wp}$ | 8.14% |

| **Atom** | **Wyckoff positions** | **Occupancy** |
|---|---|---|
| Co | 0 0 0 | 0.49 (2) |
| Mn | 0 0 0 | 0.33 (8) |
| Ni | 0 0 0 | 0.16655(4) |
| $Na_1$ | 0 0 0.25 | 0.2155(8) |
| $Na_2$ | 0.33 0.66 0.75 | 0.4834(1) |
| O | 0.33 0.66 0.096 | 0.16667 |

**Supplementary Table 1.** Crystallographic parameters of P2 $Na_{0.78}Co_{1/2}Mn_{1/3}Ni_{1/6}O_2$ refined by Rietveld analysis

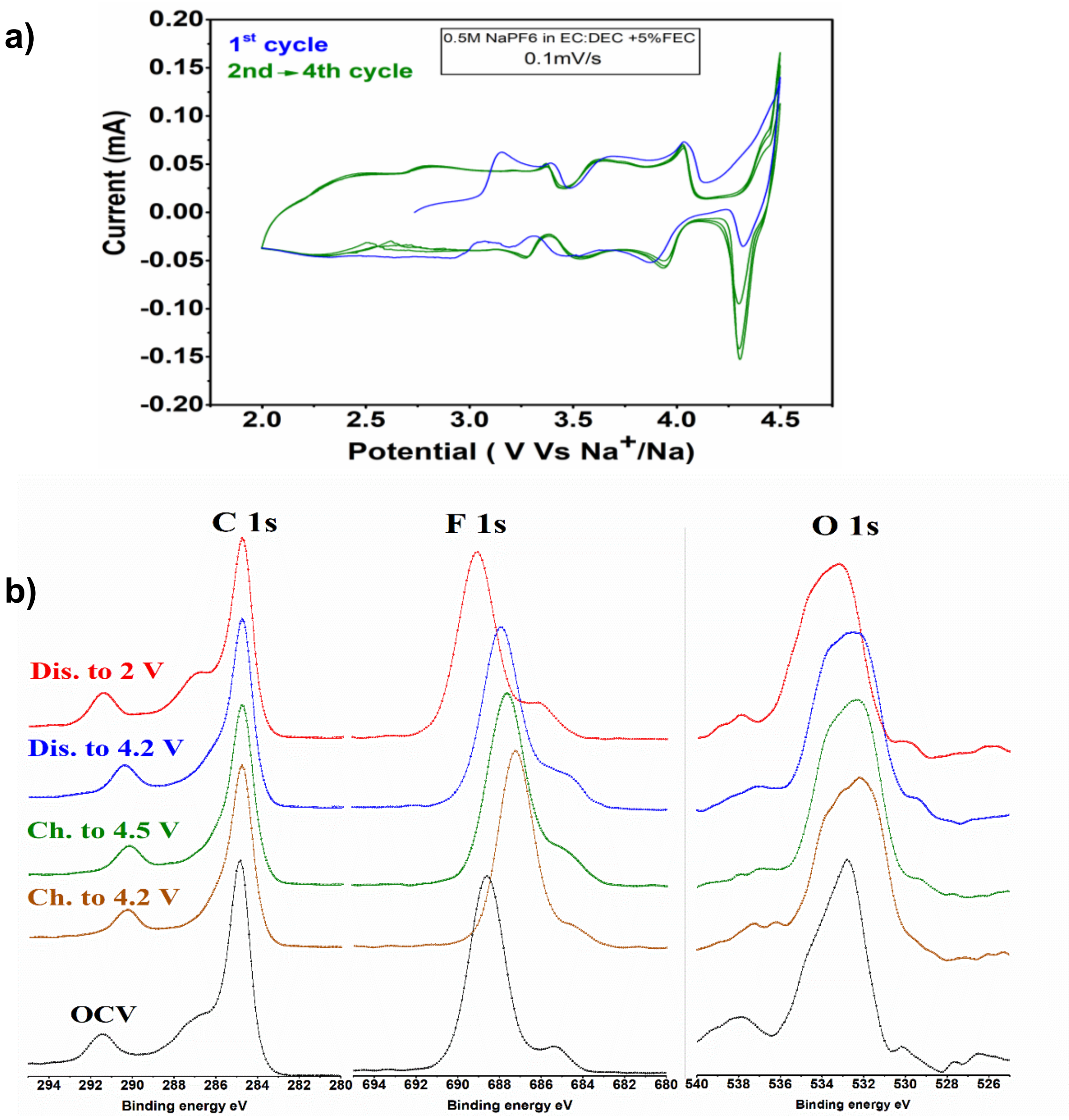


**Supplementary Figure 1.** Cyclic voltammetry and X-ray photoelectron spectroscopy results to investigate the electrolyte degradation**. a** Cyclic voltammetry of $Na_{0.78}Co_{1/2}Mn_{1/3}Ni_{1/6}O_2$ in the voltage range of [2-4.5 V] at a scan rate of 0.1mV/s. **b** C 1s, F 1s and O 1s core level XPS spectra of $Na_{0.78}Co_{1/2}Mn_{1/3}Ni_{1/6}O_2$ electrodes at different state of charges during the first cycle.

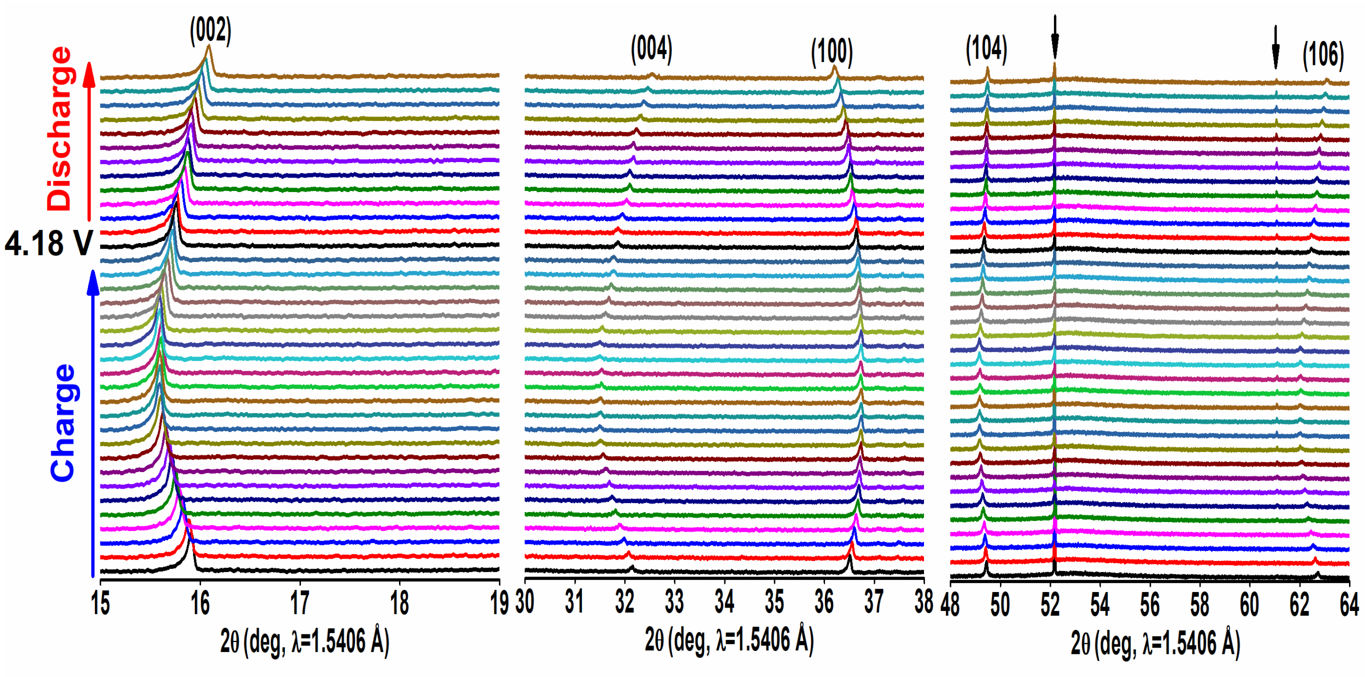


**Supplementary Figure 2.** In situ X ray diffraction patterns of P2-$Na_{0.78}Co_{1/2}Mn_{1/3}Ni_{1/6}O_2$ during the first cycle in the voltage range of 2V to 4.2 V. Peaks labelled with arrows represent reflections from the cell components.

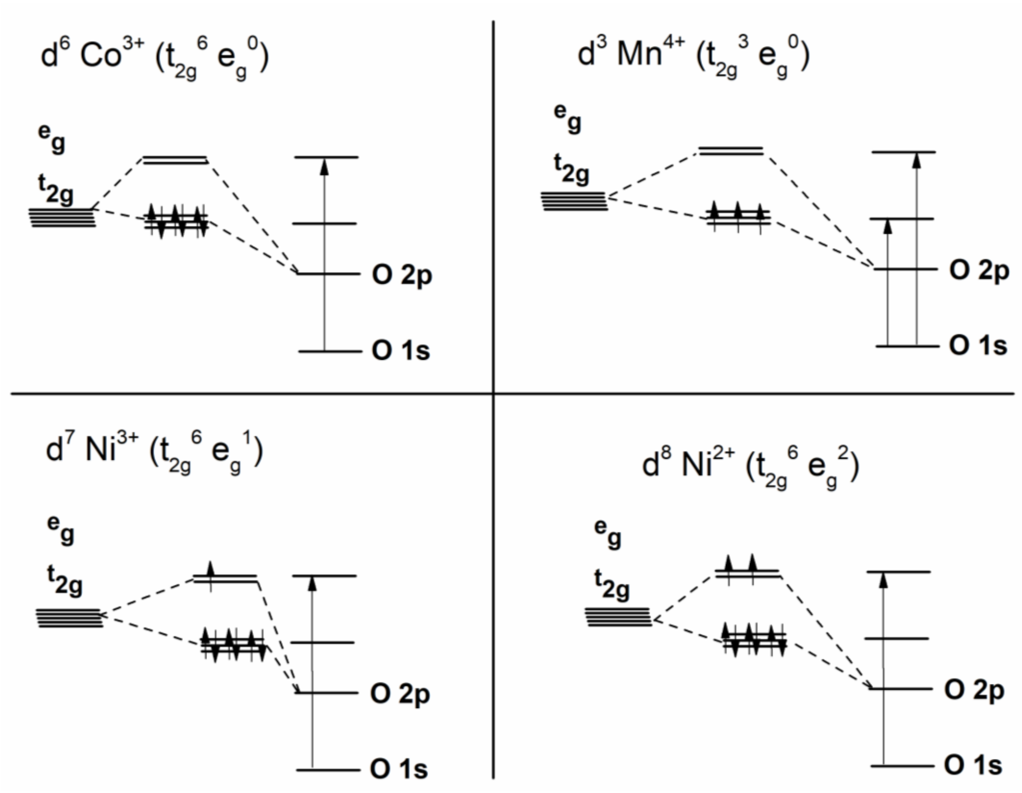


**Supplementary Figure 3.** Schematic diagrams showing the X ray absorption spectroscopy electronic transitions from the Oxygen 1s to the empty states on each of the transition metals in $Na_{0.78}Co_{1/2}Mn_{1/3}Ni_{1/6}O_2$.

**Supplementary Note 1**

The possible O K-edge SXAS transitions anticipated to these unoccupied states are given in Figure 3. There are 7 possible SXAS transitions associated with $Mn^{4+,}$ three spin-down $t_{2g}$, two spin-up and two spin-down $e_g$, with 0.33 Mn present per formula unit this implies we would expect 2.31 transitions per formula unit. For $Co^{3+}$ two spin-up and 2 spin-down $e_g$ transitions gives 2 transitions per formula unit. $Ni^{3+}$ one spin-up $e_g$ and two spin-down $e_g$ transitions which gives 0.48 transition per formula, $Ni^{2+}$ two spin-down eg transition means 0.32 transition per formula. Therefore, $Mn^{4+}$ accounts for ~45 % of the expected SXAS transitions to the unoccupied states, Co3+ for 39% and hence both Mn and Co dominate the spectrum.

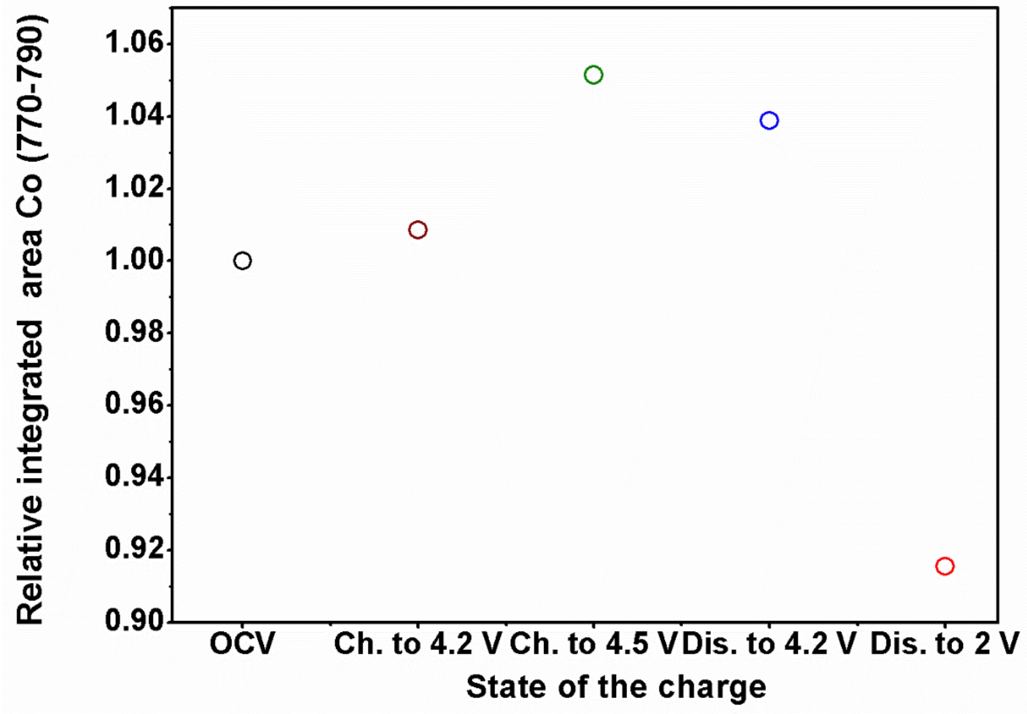


**Supplementary Figure 4.** Variation of the integrated intensity for Co L-edge SXAS
